# Deletion of *Trpm4* Alters the Function of the Na_v_1.5 Channel in Murine Cardiac Myocytes

**DOI:** 10.3390/ijms22073401

**Published:** 2021-03-26

**Authors:** Lijo Cherian Ozhathil, Jean-Sébastien Rougier, Prakash Arullampalam, Maria C. Essers, Daniela Ross-Kaschitza, Hugues Abriel

**Affiliations:** Institute of Biochemistry and Molecular Medicine, and Swiss National Centre of Competence in Research (NCCR) TransCure, University of Bern, Bühlstrasse 28, 3012 Bern, Switzerland; jean-sebastien.rougier@ibmm.unibe.ch (J.-S.R.); prakash.arullampalam@ibmm.unibe.ch (P.A.); maria.essers@ibmm.unibe.ch (M.C.E.); daniela.ross@ibmm.unibe.ch (D.R.-K.)

**Keywords:** TRPM4, SCN5A, intracardiac ECG, mexiletine, cardiac conduction disorder, channelosome

## Abstract

Transient receptor potential melastatin member 4 (TRPM4) encodes a Ca^2+^-activated, non-selective cation channel that is functionally expressed in several tissues, including the heart. Pathogenic mutants in *TRPM4* have been reported in patients with inherited cardiac diseases, including conduction blockage and Brugada syndrome. Heterologous expression of mutant channels in cell lines indicates that these mutations can lead to an increase or decrease in TRPM4 expression and function at the cell surface. While the expression and clinical variant studies further stress the importance of TRPM4 in cardiac function, the cardiac electrophysiological phenotypes in *Trpm4* knockdown mouse models remain incompletely characterized. To study the functional consequences of *Trpm4* deletion on cardiac electrical activity in mice, we performed perforated-patch clamp and immunoblotting studies on isolated atrial and ventricular cardiac myocytes and surfaces, as well as on pseudo- and intracardiac ECGs, either in vivo or in Langendorff-perfused explanted mouse hearts. We observed that TRPM4 is expressed in atrial and ventricular cardiac myocytes and that deletion of *Trpm4* unexpectedly reduces the peak Na^+^ currents in myocytes. Hearts from *Trpm4^−/−^* mice presented increased sensitivity towards mexiletine, a Na^+^ channel blocker, and slower intraventricular conduction, consistent with the reduction of the peak Na^+^ current observed in the isolated cardiac myocytes. This study suggests that TRPM4 expression impacts the Na^+^ current in murine cardiac myocytes and points towards a novel function of TRPM4 regulating the Na_v_1.5 function in murine cardiac myocytes.

## 1. Introduction

The cardiac Ca^2+^-activated non-selective cation (NSca) currents were first measured in cultured rat neonatal myocytes in the early 1980s [1]. The molecular identities of these current components remained largely unknown until a member of TRPM family, TRPM4b, was cloned [2,3,4,5], which was found to share the biophysical properties of a native NSca current from human atrial myocytes [6]. TRPM4 belongs to the transient receptor potential (TRP) ion channel superfamily, comprising mostly large Ca^2+^-permeable cation channels that are expressed in different tissue types and are activated by a broad spectrum of physicochemical stimuli under varying cellular conditions. TRPM4 and TRPM5 are the only members of the TRP family that are not permeable to divalent cations such as Ca^2+^ or Mg^2+^. Instead, both are activated by an increase in intracellular Ca^2+^, and voltage further modulates its gating, resulting in an outwardly rectifying current [7,8,9]. This distinctive biophysical feature could allow Na^+^ entry at negative potentials leading to membrane depolarization and K^+^ efflux at positive potentials facilitating membrane repolarization.

In human hearts, the expression of TRPM4 has been detected in Purkinje fibers, atria, and ventricles [10]. The clinical relevance of TRPM4 emerged from the identification of the first mutation in patients with hereditary cardiac conduction slowing disorders. This mutation caused the replacement of a glutamate residue at position 7 with lysine (p.E7K), which led to an increase in protein function and surface expression [10]. To date, more than 25 variants of TRPM4 are associated with conduction disorders and Brugada syndrome [11,12,13,14,15]. However, phenotype–genotype correlations with TRPM4 variants remain ambiguous, as both gain- and loss-of-function were reported to have overlapping clinical phenotypes. In one of our recent works, we found that some *TRPM4* mutations altered the protein degradation rate, which led to either increased or decreased stability of the protein at the membrane [13]. How the altered degradation rates related to the clinical phenotypes of the respective mutations is still unclear. In mouse hearts, the role of TRPM4 has been studied via genetic ablation of *Trpm4* [16,17] and inhibition of TRPM4-mediated current using 9-phenanthrol [18,19,20]. In both cases, the cardiac action potentials (APs) were shortened, suggesting a role of TRPM4 in the repolarization phase. However, discrepancies exist in these studies regarding their baseline cardiac phenotypes and doubts regarding the lack of specificity of 9-phenanthrol [21,22,23].

Previous studies have proposed the hypothesis that TRPM4 may functionally affect the activity of other cardiac ion channels, such as the main cardiac voltage-gated Na^+^ channel Na_v_1.5 [12,24]. Since TRPM4 carries an inward current close to resting membrane potential and an outward current at depolarized potentials, increasing or decreasing TRPM4 expression may critically influence the biophysical availability of Na_v_1.5 channels. To test this hypothesis, we compared the cardiac electrophysiology in mice where *Trpm4* was deleted and examined whether this affected Na_v_1.5 function or expression.

## 2. Results

### 2.1. Deletion of Trpm4 Affects the Mouse Cardiac Action Potential

**Figure 1:**
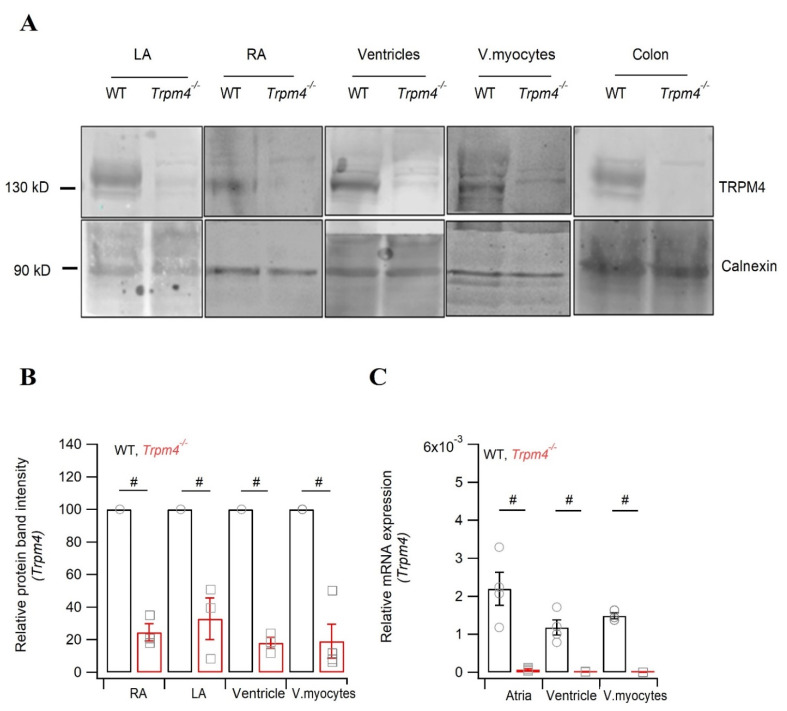
Expression of Trpm4 in mouse heart models. (**A**) Representative immunoblots of Trpm4 protein expression from right (RA) and left (LA) atrium, ventricles, isolated ventricular myocytes (v.myocytes), and colon tissue. (**B**) Quantification of the immunoblot showing protein expression in the atria (N = 10), ventricles (N = 11), and v.myocytes (*N* = 4). (**C**) Quantification of *Trpm4* mRNA using RT-qPCR. The Ct values were normalized to GAPDH (*N* = 4). Note: #: *p* < 0.05, WT (circle, black) vs. *Trpm4^−/−^* (square, red).

To investigate the role of TRPM4 on mouse cardiac electrical activity, first we evaluated the expression of TRPM4 in different compartments of the heart: the atria, ventricles, and isolated ventricular myocytes. In line with previous reported studies [16,17,25], we observed expression in the atria, ventricles, and also in isolated ventricular myocytes at both the protein (Figure 1A,B) and mRNA (Figure 1C) level. Atria showed a trend for higher TRPM4 expression than in ventricles. *Trpm4^−/−^* mice [26] showed a reduction of the 130 kDa TRPM4 signal in both atria and ventricles. Since a strong expression of TRPM4 has been previously reported in the colon, we used it as a positive control in wild type (WT) mice. Expectedly; this signal was reduced in *Trpm4^−/−^* (Figure 1A).

**Figure 2:**
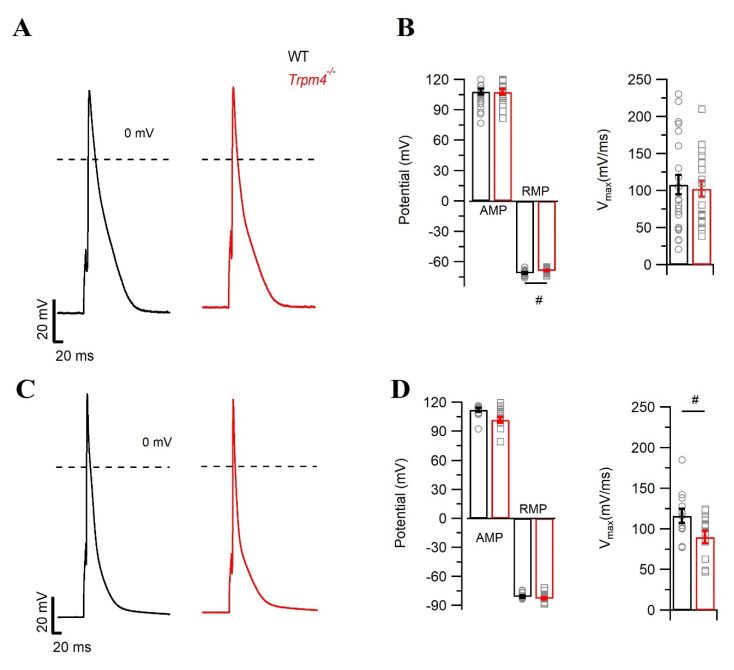
Action potential measurement in isolated mouse atrial and ventricular myocytes. Representative traces of AP recorded in WT (black) and *Trpm4^−/−^* (red) mice either from (**A**) right atrial myocytes (*N* = 5, *n* = 19) or (**C**) ventricular myocytes (*N* = 5, *n* = 14). Average values for AMP (peak amplitude), RMP, V_max_ in WT (circle, black), and *Trpm4^−/−^* (square, red) either from (**B**) right atrial myocytes or (**D**) ventricular myocytes. Note: #: *p* < 0.05, WT vs. *Trpm4^−/−^*.

To determine the functional consequences of *Trpm4* deletion on cardiac electrical activity at the cellular level, we recorded the cardiac action potential (AP) from freshly isolated right atrial and ventricular cardiomyocytes from WT and *Trpm4^−/−^* mice (Figure 2). In AP recordings from atrial cardiomyocytes, we did not observe any difference in upstroke velocity (V_max_) or action potential duration (APD) (Table 1) among the groups (Figure 2A,B). However, the resting membrane potential (RMP) of atrial cardiomyocytes from *Trpm4^−/−^* was significantly depolarized by ~1 mV when compared to the WT mice (Figure 2B). Since in cardiac myocytes, inward rectifier current (*I*_K1_) is one of the major components for the RMP, we compared the *I*_K1_ currents and did not observe any significant alterations in the peak currents (*p* > 0.05, Supplementary Figure S1). We also measured APs in ventricular cardiomyocytes (Figure 2C). Here, we observed a decrease in the V_max_ in *Trpm4^−/−^* mice (*p* = 0.03), whereas neither APDs nor the RMP levels were altered (Figure 2D and Table 1).

### 2.2. Deletion of Trpm4 Affects Na^+^ Currents in Atrial and Ventricular Myocytes

**Figure 3:**
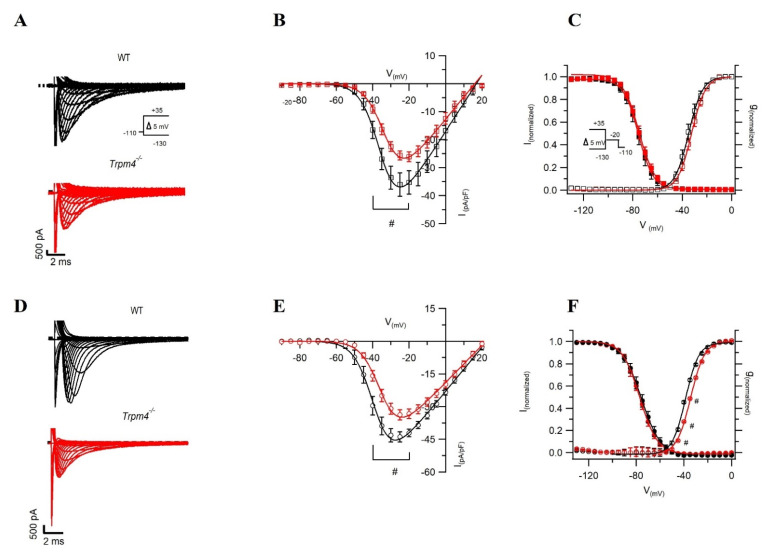
Na^+^ current recordings from isolated cardiomyocytes. Representative Na^+^ current traces recorded from myocytes in WT (black) or *Trpm4^−/−^* (red) mice, either from ventricular (*N* = 5, *n* = 9 WT, *n* = 12 *Trpm4^−/−^*) (**A**) or atrial (*N* = 5, *n* = 20 WT, *n* = 22 *Trpm4^−/−^*) tissue (**D**), using a voltage step protocol as shown in inset. Na^+^ current–voltage relationships in WT and *Trpm4^−/−^* mice from either ventricular (**B**) or atrial (**E**) cardiomyocytes. (**C**,**F**) Voltage dependence of activation (open square or circle) and inactivation (filled in square or circle) fitted with Boltzmann equation in WT and *Trpm4^−/−^* mice from either ventricular or atrial cardiomyocytes, respectively. Note: #: *p* < 0.05, WT vs. *Trpm4^−/−^* at a given voltage).

To study the ionic mechanism contributing to the decrease in the V_max_ of the action potential recordings from ventricular myocytes, we measured Na^+^ currents using a voltage step protocol in whole-cell configuration. In ventricular cardiomyocytes, the peak Na^+^ current (*I_Na_*) density was reduced by 30% in *Trpm4^−/−^* mice compared to WT mice (Figure 3A,B and Table 2). Although our AP recordings from atrial myocytes did not reveal any alterations in the upstroke velocity, the peak Na^+^ current in atrial myocytes was reduced by 25% in *Trpm4^−/−^* compared to WT mice (Figure 3D,E and Table 2). In addition, the membrane capacitance of atrial myocytes from *Trpm4^−/−^* was reduced by 20% compared to the WT condition (Table 2 and Supplementary Figure S1B). Nevertheless, the decrease in peak Na^+^ current observed in atrial myocytes from *Trpm4^−/−^* mice was not a consequence of the reduced membrane capacitance, as the current density and the current both showed a similar reduction (Supplementary Figure S2).

To further assess any alterations in the biophysical properties of the peak Na^+^ current, we measured the *V*_1/2_ levels of steady-state activation and inactivation. We observed no differences in *V*_1/2_ levels of steady-state inactivation and activation between WT and *Trpm4^−/−^* ventricular cardiomyocytes (Figure 3C and Table 2). In atrial myocytes, however, we observed a significant depolarizing shift of 4 mV in the *V*_1/2_ of steady-state activation in *Trpm4^−/−^* mice compared to WT mice, while the steady-state inactivation was not altered (Figure 3F and Table 2).

Since this reduction of the peak Na^+^ current was not previously reported in this mouse line [16], we recently generated in our laboratory a new global knockdown of the *Trpm4* mouse strain (called B.*Trpm4^−/−^*) on a C57BL/6J background by targeting *Trpm4* exon 10 instead of the exon 15–16 (Supplementary Figure S3) [26], which resulted in a decrease in TRPM4 expression in B.*Trpm4^−/−^*(Supplementary Figure S4). We measured the peak Na^+^ current in WT and *Trpm4^−/−^* ventricular myocytes and observed a similar decrease of 25% in the peak Na^+^ current in these B. *Trpm4^−/−^* mice (Supplementary Figure S5).

### 2.3. Trpm4^−/−^ Mouse Hearts Display an Increased Sensitivity towards the Na^+^ Channel Inhibitor Mexiletine

Since our data indicated alterations in Na^+^ current densities in atrial and ventricular *Trpm4^−/−^* cardiomyocytes, we next investigated the consequences of *Trpm4* deletion on the whole heart using in vivo surface electrocardiograms (ECGs) in deeply anesthetized mice. We did not observe any significant alteration in the ECG parameters, aside from the heart rate (HR) (Table 3). The heart rate in *Trpm4^−/−^* was markedly reduced to 280 ± 19 bpm (N = 7) compared to WT mice (361 ± 20 bpm) (*N* = 4).

**Figure 4:**
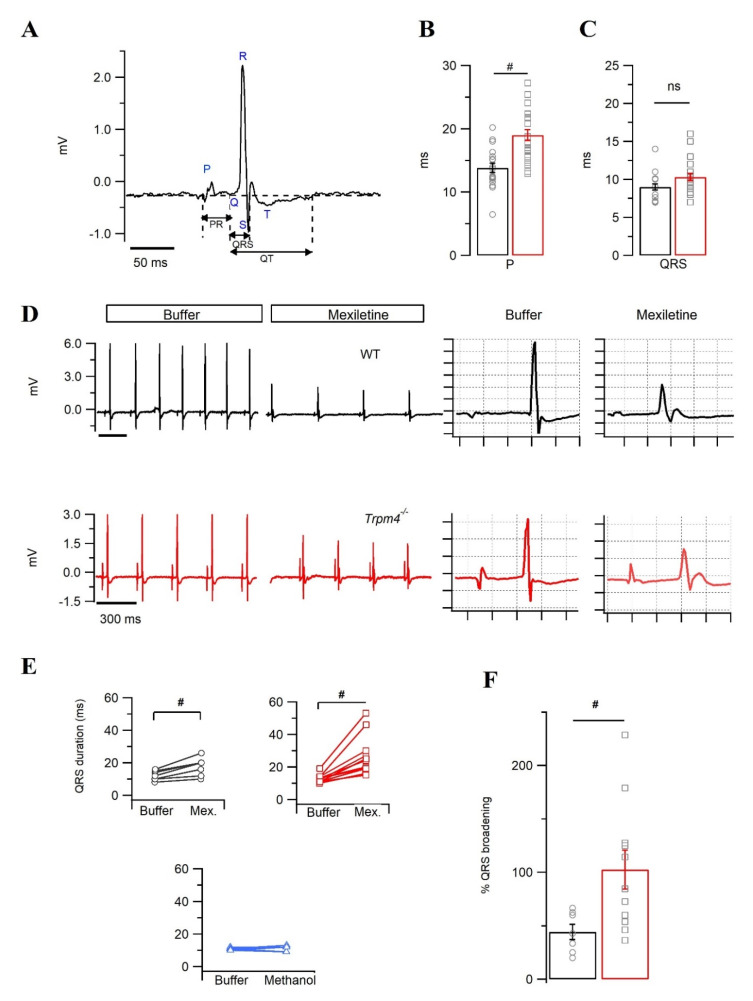
Pseudo-electrocardiogram recordings of WT and *Trpm4*^−/−^ explanted hearts. (**A**) Representative pseudo-ECG trace from WT heart highlighted for different ECG parameters, considered for further comparative studies. Average P (**B**) and QRS (**C**) durations recorded from either WT (*N* = 19: circle, black) or *Trpm4*^−/−^ (*N* = 22: square, red) hearts. (**D**) Representative ECG traces before and after perfusion with mexiletine. (**E**) QRS durations calculated when perfused with either buffer or mexiletine (Mex.) in WT (*N* = 7, black) and *Trpm4^−/−^* (*N* = 11, red) mice or perfused with the vehicle control, methanol (*N* = 6, blue). (**F**) The degree of broadening of QRS due to mexiletine perfusion compared between the genotypes as % inhibition (WT: circle, black and *Trpm4*^−/−^: square, red). Note: #: *p* < 0.05, WT vs. *Trpm4^−/−^*, ns- not significant.

Further investigations of the electrical properties of *Trpm4^−/−^* mice were conducted using pseudo-ECG recordings on Langendorff-perfused, explanted mouse hearts. Such explanted mouse hearts allow perfusion of different drugs, while alterations in electrical properties can be monitored in real-time. Figure 4A shows an example of a two-lead pseudo-ECG and the analyzed parameters on a perfused, explanted WT mouse heart beating spontaneously. In pseudo-ECGs recorded from control buffer perfused hearts, the P duration was significantly increased in *Trpm4^−/−^* compared to WT mice (*p* = 6 × 10^−5^) (Figure 4B and Table 3). Other ECG parameters, including the PR interval, QT, QTc, and HR, were not altered by *Trpm4* deletion. QRS duration, however, showed a non-significant trend for broadening in *Trpm4^−/−^* hearts (*p* = 0.06) (Figure 4C and Table 3).

The tendency of the QRS complex to be prolonged in pseudo-ECGs and the decreased *I_Na_* observed in *Trpm4^−/−^* cardiac myocytes suggest that the decrease of sodium current may lead to QRS prolongation in *Trpm4^−/−^* hearts. To investigate this hypothesis, we decided to challenge the explanted hearts with 40 µg/mL mexiletine, a Na^+^ channel blocker (Figure 4D) [27]. Mexiletine broadened the QRS duration in both genotypes (Figure 4E). However, the QRS duration from *Trpm4^−/−^* hearts presented a two-fold increase in mexiletine sensitivity compared to WT hearts (QRS broadening (%): 102 ± 18 vs. 44 ± 7, *p* = 0.02) (Figure 4F). The QRS broadening observed in our recordings was exclusively mexiletine-dependent, as methanol (vehicle control) did not have any effect on the QRS duration (Figure 4E).

### 2.4. Intraventricular Conduction Is Slower in Trpm4^−/−^ Mouse Hearts Than in WT Mouse Hearts

**Figure 5:**
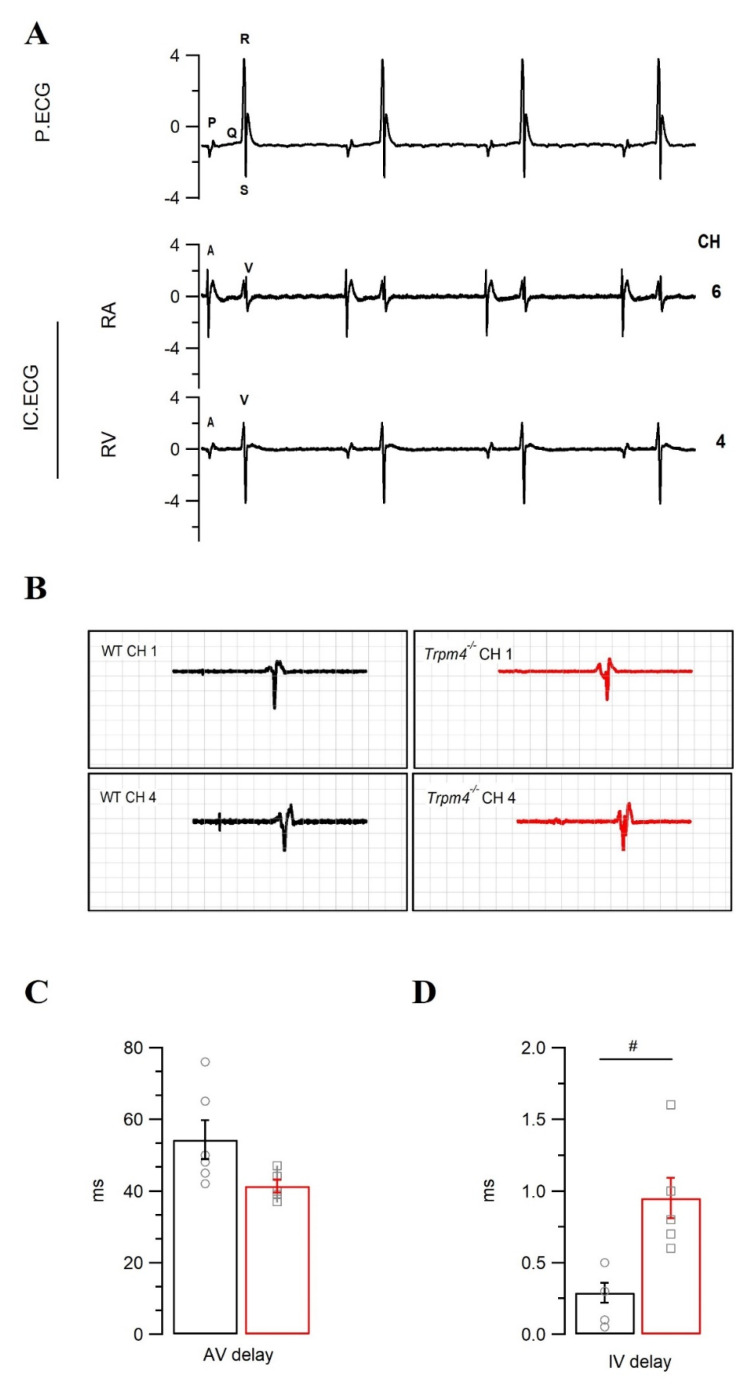
Intracardiac electrocardiogram recordings on WT and Trpm4^−/−^ explanted hearts. (**A**) Representative pseudo (P.ECG) and intracardiac (IC.ECG) ECG trace from a WT heart. A and V respectively represent atrial and ventricle signals from the intracardiac catheter recorded in different electrode channels (CH 1-7). Conduction delays either between the atria and ventricle (AV delay, CH 6-5) or intraventricular (IV delay, CH 4-1) were measured as the time delay between respective channel signals. Representative trace showing signal derivatives from CH 1 and 4 to measure IV delay (**B**). Average AV delay (**C**) and IV delay (**D**), compared between the genotypes (WT: circle, black and *Trpm4*^−/−^: square, red). Note: *N* = 6, #: *p* < 0.05, WT vs. *Trpm4*^−/−^; ns = not significant.

To investigate any potential conduction delay due to the reduction of the peak Na^+^ current in *Trpm4^−/−^* mice, we performed intracardiac ECG measurements on the spontaneously beating explanted hearts using intracardiac catheters with eight electrodes. In most explanted hearts, we could acquire electrical activity readings from at least six electrodes, one from the right atrium and five from the right ventricle. Pseudo-ECGs were simultaneously acquired, allowing the correct identification of atrial (A) and ventricular (V) peaks in intracardiac ECGs (Figure 5A). The AV delay measured between the A and V signals at an electrode distance of 1 mm did not reveal any difference between the genotypes (Figure 5C). However, the intraventricular (IV) conduction time from the right ventricle measured at an electrode distance of 3 mm was markedly slower in *Trpm4^−/−^* than in WT hearts (0.95 ± 0.1 vs. 0.3 ± 0.07 ms, *p* = 0.002) (Figure 5B,D). Since we observed conduction delays in *Trpm4^−/−^* mice ventricles, we also checked for the expression of connexin 43 (Cx 43), an important gap junction protein for ventricular conduction [28]. As shown in Supplementary Figure S6***,*** we did not observe any alteration in Cx 43 protein or mRNA expression due to *Trpm4* deletion.

### 2.5. Knockdown of Trpm4 Does Not Alters Na_v_1.5 Expression in Mouse Ventricles

**Figure 6:**
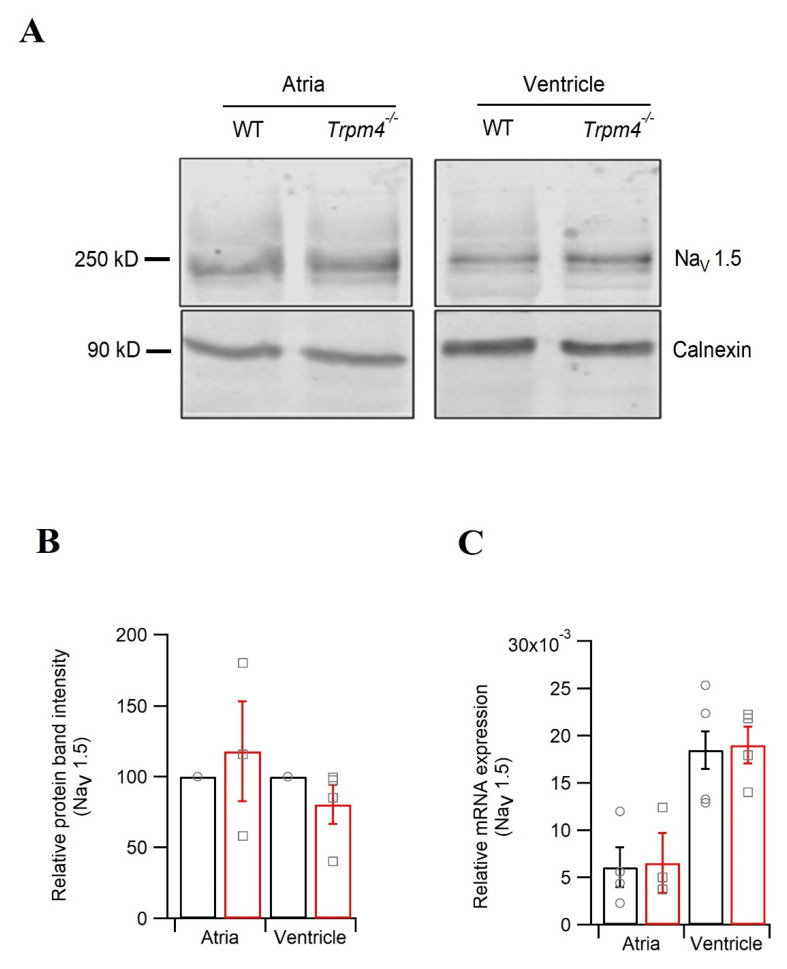
Expression of Na_v_1.5 in mouse atria and ventricles. (**A**) Representative immunoblots showing Na_v_1.5 expression from atria and ventricles, either from WT or *Trpm4^−/−^* mice. (**B**) Quantification of the immunoblots showing the total Na_v_1.5 expression in atria (*N* = 6; *p* = 0.63) and ventricles (*N* = 13; *p* = 0.20): WT (circle, black) or *Trpm4^−/−^* (square, red). (**C**) Na_v_1.5 mRNA quantification using RT-qPCR from atrial and ventricular tissue; WT (circle, black) or *Trpm4^−/−^* (square, red). The Ct values were corrected with GAPDH (*N* = 4; *p* = 0.87 and 0.89 for atria and ventricles, respectively).

Since we observed decreases in the peak Na^+^ current in atrial and ventricular myocytes, and our ECG studies further confirm reduced Na^+^ currents in *Trpm4^−/−^* hearts, we assessed the Na_v_1.5 expression in both chambers of the heart. Western blot analysis of the total Na_v_1.5 protein (Figure 6A,B) or mRNA expression of *Scn5a* encoding Na_v_1.5 (Figure 6C) remained unchanged between WT and *Trpm4^−/−^* mice.

### 2.6. Na_v_1.5 Interacts with TRPM4 in an Heterologous Expression System

**Figure 7:**
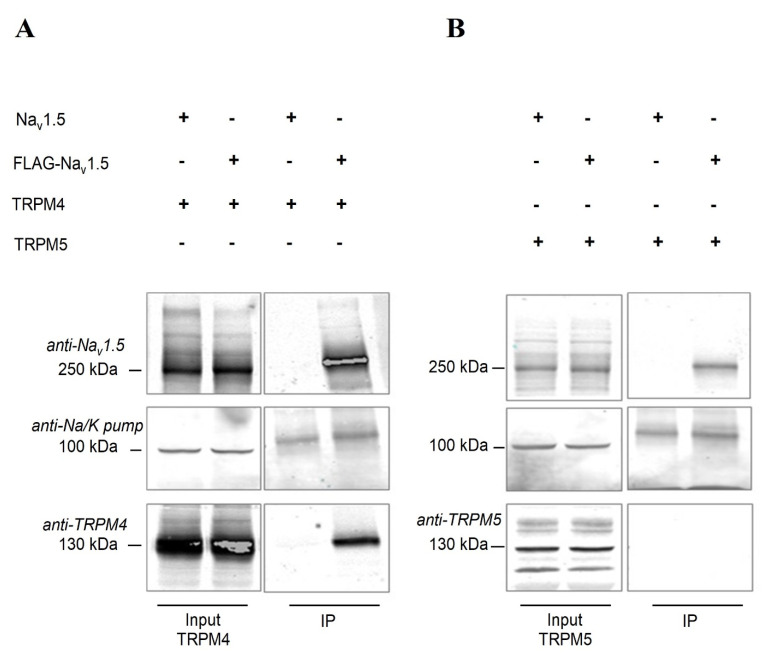
Co-immunoprecipitation of Na_v_1.5 and TRPM4 in HEK-293 cells. (**A**) Left panel: Input signals confirming the expression of Na_v_1.5 with or without FLAG tag and TRPM4. The alpha subunit of the Na/K pump was used as a loading control. Right panel: In the immunoprecipitated (IP) fraction, the signal was observed only in the presence of FLAG-Na_v_1.5 and TRPM4. (**B**) Left panel: Input signals confirming the expression of Na_v_1.5 with or without FLAG tag and TRPM5. Right panel: In the immunoprecipitated fraction, no signal was observed for TRPM5, even though the IP of FLAG-Na_v_1.5 was successful.

Electrophysiological studies on myocytes from atria and ventricles showed a 20% reduction of the peak Na current in *Trpm4^−/−^* mice, however we could not corroborate it to a reduction of Na_v_1.5 expression in the atrial or ventricular tissue from *Trpm4^−/−^* mice. Therefore, we set out to study if TRPM4 and Na_v_1.5 proteins interact. Human embryonic kidney (HEK-293) cells were transiently co-transfected with FLAG-tagged human-Na_v_1.5 (FLAG-Na_v_ 1.5) and human-TRPM4, and co-immunoprecipitation was performed to evaluate if any interaction exists between these two proteins. As shown in Figure 7, FLAG-Na_v_1.5 was immunoprecipitated (IP) and TRPM4 was co-immunoprecipitated (Figure 7A). Co-immunoprecipitation of TRPM4 and Na_v_1.5 was confirmed in HEK293 in 4 different biological replicates (Supplementary Figure S7). As shown in Figure 7, we observed a signal for the sodium potassium (Na/K) pump in the IP fraction, which may have been due to non-specific binding of the Na/K pump to the dynabeads. To confirm the specificity of TRPM4–Na_v_1.5 interaction, we used the closest analog of TRPM4, TRPM5, and no longer observed the interaction with Na_v_1.5 (Figure 7B). Furthermore, we also ruled out any non-specific interaction between Na_v_1.5 and TRPM4 by including a non-FLAG-tagged Na_v_1.5 plasmid. Here, we did not observe any signal for either Na_v_1.5 or TRPM4 in the IP fraction.

## 3. Discussion

In the present study, we compared the functional expression of TRPM4 in freshly isolated murine atrial and ventricular myocytes and studied the consequences of *Trpm4* deletion on mouse heart electrical activity. We observed an unexpected decrease in Na_v_1.5 function in *Trpm4*-deficient mouse hearts. Therefore, we were interested to find out if Na_v_1.5 and TRPM4 do interact and how they modulate the cardiac electrical activity.

Since the reports in which TRPM4 was identified as the prime molecular candidate for NSca [2,4], functional expression of this channel has been detected in different parts of the heart, such as the sino-atrial node, atria, and at lower levels in ventricles [6,11,17,20,25,29]. In our study, we observed TRPM4 signals in the atria, ventricles, and isolated ventricular myocytes in WT mice, which were absent in *Trpm4^−/−^* mice.

The functional role of this channel in cardiac electrical activity remains unclear. TRPM4 is suggested to prolong APDs [16,18], which was shown using the generic TRPM4 inhibitor 9-phenanthrol [19,30,31] and in *Trpm4^−/−^* mice [16,17]. In our perforated-patch AP recordings in isolated myocytes, we did not observe any changes in the APDs; however, the upstroke velocity in *Trpm4^−/−^* ventricular myocytes was significantly slower compared to those in WT mice. The fast kinetics of Na_v_1.5 underlie the upstroke velocity of a cardiac AP. We, therefore, compared any alteration in the functional expression of Na_v_1.5 in atria and ventricle due to *Trpm4* deletion. Indeed, the peak Na^+^ currents conducted by Na_v_1.5 in cardiac myocytes were reduced by 25 and 30% respectively in atria and ventricles of *Trpm4^−/−^* mice compared to WT mice. However, the decreases in the peak Na current did not correlate with the Na_v_1.5 expression, as we could not observe any alterations in the total Na_v_1.5 protein or mRNA expression in atrial or ventricular tissue in either our Western blot or mRNA quantification from *Trpm4^−/−^* mice hearts. Based on this unexpected observation from Na_v_1.5 expression studies, we were interested in examining whether TRPM4 and Na_v_1.5 proteins interact. Indeed, the co-immunoprecipitation studies conducted in HEK-293 cells transiently co-transfected with TRPM4 and Na_v_1.5 plasmid showed an interaction, which was absent when Na_v_1.5 was co-transfected with TRPM5.

Furthermore, in atria where ours and others study reported stronger TRPM4 expression compared to ventricles [16,17,18,25], the impact of *Trpm4* deletion is more complex. Along with decreases in the peak Na^+^ current, the biophysical properties of the Na^+^ current were also altered, with a depolarizing shift of 4 mV in steady-state activation. The RMP of atrial myocytes from *Trpm4^−/−^* mice was depolarized by ~1 mV; however, we did not observe any alteration in *I*_K1_ in atrial myocytes from *Trpm4^−/−^* mice. The combined effects of reduced peak Na^+^ currents, altered biophysical properties, and depolarized RMP in *Trpm4^−/−^* could reduce the total availability of Na_v_1.5 for activation in atria. Combining these cellular studies in atria, ventricles, and heterologous expression systems suggests that TRPM4 is an interacting partner of Na_v_1.5; how this interaction leads to regulation of Na_v_1.5, particularly reduction of *I_Na_* in the absence of TRPM4, remains to be further studied.

The Na_v_1.5-mediated Na^+^ current plays a critical role in excitability and conduction velocity in cardiac tissue [32,33]. The strong functional reduction of Na_v_1.5 in atrial and ventricular cardiac myocytes observed in our study was expected to be reflected in the whole-heart electrical activity by slowing down atrial and ventricular depolarization [34]. Indeed, in our pseudo-ECG recordings of explanted hearts under basal conditions, the P durations were longer and QRS durations showed a trend toward broadening in *Trpm4^−/−^* hearts. Challenging the explanted hearts with the Na^+^ channel blocker mexiletine confirmed the broadening of QRS in *Trpm4^−/−^* hearts, which were more sensitive towards mexiletine than WT hearts. In addition to slower depolarization, *Trpm4^−/−^* hearts also presented significant slowing of intraventricular conduction in the right ventricles compared to those in WT mice, as observed in intracardiac electrocardiograms. We speculate that this could mainly be the consequence of reduced Na_v_1.5 function, because Cx 43 expression, another key protein involved in electrical conduction propagation in the heart, was not altered in *Trpm4^−/−^* ventricles. Given their small size, we were not able to measure conduction velocity in atria. Future studies using perfused atrial tissue preparation and quantifying Cx 40/43 expression are clearly warranted. Nonetheless, by itself, the functional reduction of Na_v_1.5 caused by the *Trpm4* deletion observed both in atrial and ventricular myocytes most likely suffices to slow conduction in *Trpm4^−/−^* mice hearts.

In addition to slower conduction in cardiac tissue, surface ECGs from anesthetized *Trpm4^−/−^* mice showed lower heart rates than those from WT mice. Although a direct decrease in heart rate was not observed in the previous studies, the slowing of electrical propagation and sinus pauses were more evident in *Trpm4^−/−^* mice than in WT mice [17]. Moreover, in this study [17], the mice moved freely and the baseline heart rate was ~500 bpm, while in our case under anesthesia, the heart rate was ~300 bpm. This somehow unmasked the effects of *Trpm4* deletion on the heart rate. A previous study [35] showed that in the sinoatrial node (SAN) in mice, TRPM4 activates at lower heart rates, thus acting as an accelerator and increasing the heart rate to counteract bradycardia. However, we did not observe any heart rate change in spontaneously beating, explanted, denervated *Trpm4^−/−^* hearts in our pseudo-ECGs, where the basal heart rate was even lower (~220 bpm) (Table 2). These findings raise an intriguing question of whether the alterations in heart rate due to *Trpm4* deletion were a direct effect of SAN electrical dysfunction or were due to autonomic regulation. Interestingly, recent studies have highlighted the functional relevance of TRPM4 in neurons of the brainstem [17,36] and in the release of neurotransmitters in the autonomic ganglia [25], which could also implicate TRPM4 in regulating the heart rate via the autonomic nervous system. The functional consequence of *Trpm4* deletion on the heart rate is still unclear and demands further study.

Besides altered Na_v_1.5 function and slowing down of the heart rate, *Trpm4* deletion also reduced the cell surface area, as the membrane capacitance of atrial myocytes from *Trpm4^−/−^* hearts was found to be significantly reduced. A previous study reported a similar decrease in the cell surface area of ventricular myocytes from *Trpm4^−/−^*, with an increase in cellular density [17]. The authors reported left ventricular hypertrophy due to hyperplasia during the proliferative stage in *Trpm4^−/−^* mice. We have not evaluated any existence of a similar hypertrophy due to hyperplasia in *Trpm4^−/−^* mouse atrial or ventricular tissue; future studies looking into the role of *Trpm4* in cardiac proliferation in the neonatal stage are needed.

Cardiac phenotypes in *Trpm4^−/−^* mice were previously characterized earlier by different groups; none of these studies observed any alteration in peak Na^+^ currents or Na_v_1.5 protein expression [16,17]. This discrepancy between our study and other studies could be partially explained by differences in study designs used (isolated myocytes vs. whole tissues, perforated-patch clamp vs. microelectrode or whole-cell recordings). In addition, a recent review [37] highlighted that the differences in cardiac phenotype observed in *Trpm4^−/−^* mice could be mouse-line dependent. However, in our study, we showed a similar reduction of the peak Na^+^ current due to *Trpm4* deletion in ventricular myocytes from two different mouse lines that were generated independently (Figure 3 and Supplementary Figure S5). Furthermore, the altered conduction properties in whole-heart models using pseudo-ECGs on mexiletine-perfused explanted hearts were consistent with the observed reduction in peak Na^+^ current.

To the best of our knowledge, this study provides the first evidence of a functional interaction between TRPM4 and Na_v_1.5 in cardiac tissue. Na_v_1.5 contributes to the fast upstroke in the depolarization phase of the cardiac action potential, while the TRPM4-mediated current has been found in the repolarization phase [16,17,18,19]. It is intriguing how seemingly distinct depolarizing and repolarizing current components can interact. A similar concept of ion channel co-regulation has been observed recently for Na_v_1.5 and Kir2.1 [38,39,40]. The authors showed the Na_v_1.5 and Kir2.1 modulate each other’s functions and expression within a macromolecular complex through their respective PDZ-binding domains to regulate cardiac excitability. Another study showed the interaction between hERG and Na_v_1.5, even at the level of mRNA transcripts [41]. The authors demonstrated an interaction of the transcripts of hERG1a, hERG1b, and Na_v_1.5, which regulate each other’s expression at the membrane level. Although in our study we observed a functional interaction between TRPM4 and Na_v_1.5 protein, we could not confirm any alterations in total protein or mRNA expression in cardiac tissue.

Tight regulation of Ca^2+^ and Na^+^ ions is crucial for cardiac conduction, which otherwise could predispose the heart to arrhythmia. As TRPM4 is a calcium-activated channel that regulates Na_v_ function, it is strongly emphasized that any imbalance in function or expression of TRPM4 could be arrhythmogenic. Indeed, mutations in human *TRPM4* are linked to conduction disorders, such as right bundle branch blockage, Brugada syndrome, and atrioventricular blockage [24,37]. Although the first mutation reported in *TRPM4* was a gain-of-function mutation linked to progressive heart blockage, several later studies reported both gain- and loss-of-function mutations in *TRPM4* related to different cardiac conduction disorders [11,12,13,42]. Until now, the mechanisms underlying the genotype–phenotype correlations in *TRPM4* mutations have remained unclear, mainly because all the studies until now have focused mainly on the functional aspect of TRPM4 alone. Our study, suggesting (1) a physical interaction between TRPM4 and Na_v_1.5 and (2) a functional effect on the Na_v_1.5 current when this physical interaction is not possible, brings a newer perspective on the possible implications of different *TRPM4* mutations found in humans with cardiac conduction disorders. We propose that alterations in TRPM4 expression due to mutations could critically affect the function of Na_v_1.5 at the membrane level, leading to compromised cardiac excitability and conduction, which is indeed observed in *TRPM4* mutation carriers. Thus, one can speculate that the observed overlapping clinical phenotype found in patients with mutations in either *SCN5A* or *TRPM4* may be explained by the co-regulation of Na_v_1.5 function by TRPM4.

## 4. Limitations

Due to technical limitations (antibody specificity and co-immunoprecipitation on isolated cardiac myocytes), we have TRPM4 and Na_v_1.5 interactions in an overexpressed system. It will be important now to identify such a coupling of TRPM4 and Na_v_1.5 in native cardiac myocytes and their cellular distributions using imaging or biochemical tools. Nevertheless, our functional and biochemical studies on isolated atria and ventricular myocytes, whole-heart models, and heterologous expressed cellular systems are consistent with functional coupling between TRPM4 and Na_v_1.5 in mouse heart models.

In summary, we observed a physical interaction between TRPM4 and Na_v_1.5 and a *Trpm4*-deficiency-dependent peak *I_Na_* reduction in cardiac tissue, which resulted in reduced excitability and conduction. Future research identifying the molecular players in the TRPM4 and Na_v_1.5 interaction and its role in physiology and pathophysiology is critical to understand the functional role of such ion channel interactome in cardiac electrical activity.

## 5. Materials and Methods

### 5.1. Trpm4^−/−^ C57BL6/N Mice

The *Trpm4*^−/−^ mouse model is a global knockdown of the *Trpm4* gene due to the excision of exons 15 and 16, which have been previously characterized [26]. These were kind gift from Dr. Rudi Vennekens (KU Leuven) and were backcrossed for 10 generations on a C57BL/6N background. For all experiments, male *Trpm4*^−/−^ mice and wildtype (WT) littermates aged 12–15 weeks were used.

### 5.2. Isolation of Atrial and Ventricular Myocytes

Mice were anesthetized with 200 mg/kg ketamine and 20 mg/kg xylazine i.p. and sacrificed by cervical dislocation, after which the heart was rapidly excised, cannulated, and mounted on a Langendorff column for retrograde perfusion at 37 °C. The heart was rinsed free of blood for 5 min with nominally Ca^2+^-free solution containing (mmol/L): 135 NaCl, 4 KCl, 1.2 MgCl_2_, 1.2 NaH_2_PO_4_, 10 HEPES, 11 glucose, pH 7.4 (NaOH). Next, the heart was perfused with the same solutions with 50 µM Ca^2+^ and collagenase type II (1 mg/mL, CLS-2, Worthington, NJ, USA) for 15 min. Following digestion, the atria and ventricles were separated and transferred to nominal 100 µM Ca^2+^ solution. For atrial myocyte isolation, only the apex of the right atrium was excised and minced into small pieces. The cells were dispersed by gentle pipetting with a fire-polished glass pasteur pipette. The ventricle was transferred to the above mentioned buffer with 100 µM Ca^2+^, minced into small pieces to liberate single ventricular myocytes by gentle pipetting, and filtered through a 100 µm nylon mesh. For electrophysiology studies, isolated myocytes were washed 3 times and the calcium concentration was progressively increased to 1 mmol/L within ~30 min. Then, the cell suspension was placed on a gently rotating shaker at room temperature (22–25 °C) until use, within 6 h after isolation.

### 5.3. Single-Cell Myocyte Electrophysiology

#### 5.3.1. Data Acquisition and Analysis

Patch-clamp experiments were performed on isolated myocytes in voltage- or current-clamp mode using MultiClamp 700B and VE-2 controlled by Clampex 10 via a Digidata 1332A or 1440A series instrument, respectively. Data were exported to IGOR PRO (WaveMetrics, Lake Oswego, OR, USA) for analysis. Patch electrodes were pulled from borosilicate glass capillaries (World Precision Instruments, Germany GmbH) and had a resistance range of 2–5 MΩ when filled with internal solutions. All experiments were conducted at room temperature (22–25 °C).

#### 5.3.2. Action Potential Measurements

Action potential (AP) recordings on isolated myocytes were performed in perforated patch configuration. In atrial preparation, to avoid any contamination from nodal cells, we selected only atrial myocytes with rod-like and elongated shapes and with no spontaneous action potential. Cardiomyocytes were bathed in a solution containing (mmol/L): 140 NaCl, 5.4 KCl, 1.8 CaCl_2_, 1.2 MgCl_2_, 10 HEPES, 5 glucose, pH 7.4 (NaOH). Intracellular patch electrodes were filled with (mmol/L): 120 KCl, 1.5 CaCl_2_, 5.5 MgCl_2_, 5 Na_2_ATP, 5 K_2_-EGTA, 10 HEPES and amphotericin B (225 µg/mL), pH 7.4 (KOH). To evoke an action potential, a rectangular pulse of 5 ms with incremental current was injected until an AP was observed, then later 125% of this threshold current was injected at 0.5 Hz in current-clamp mode in repeated sequences for acquiring AP traces. The APs were analyzed offline to assess resting membrane potential (RMP); maximal upstroke velocity (V_max_); and 30%, 50% and 90% repolarization durations (APD). Data from 30 consecutive APs were averaged for each cell.

#### 5.3.3. Voltage Clamp Recordings

Peak sodium currents (*I*_Na_) were measured in whole-cell configuration using an internal solution (mmol/L): 60 CsCl, 70 Cs-Aspartate, 1 CaCl_2_, 1 MgCl_2_, 10 HEPES, 11 EGTA and 5 Na_2_ATP, pH 7.2 (CsOH). Isolated myocytes were bathed in solution containing (mmol/L): 10 NaCl, 120 *N*-methyl-d-glutamine (NMDG-Cl), 1.8 CaCl_2_, 1.2 MgCl_2_, 5 CsCl, 10 HEPES, 5 glucose, pH 7.4 (CsOH), along with 10 µM CoCl_2_ and 10 µM nifedipine to inhibit Ca^2+^ currents. Peak *I_Na_* was measured from a holding potential of −110 mV following steps of 5 mV from −130 to +35 mV, with a cycle length of 5 s. Current densities (pA/pF) were calculated by dividing the peak current amplitude by cell capacitance. The series resistance and cell membrane capacitance were compensated for 80%. The voltage dependence of activation was determined from the I/V relationship by normalizing peak *I_Na_* to the driving force and plotting the normalized conductance vs. membrane voltage (*V*_m_). The voltage dependence of steady-state inactivation was obtained by plotting the normalized peak current (25 ms test pulse to −20 mV after a 500 ms conditioning pulse) vs. *V*_m_. The voltage dependence levels of activation and inactivation curves were fitted with the Boltzmann function ((*V*_m_) = 1/(1 + exp[(*V*_m_ − *V*_1/2_)/*k*])), where *V*_1/2_ is the half-maximal voltage of (in)activation and *k* is the slope factor. *I*_K1_ was measured using the same internal and external solutions as those used for action potential recordings mentioned above, without amphotericin B. Na^+^ channels were blocked with 50 µM tetrodotoxin (TTX) and Ca^2+^ channels were blocked with 3 mM cobalt chloride (CoCl_2_) added to the external solution. *I*_K1_ barium-sensitive current was calculated by subtracting the potassium current recorded after perfusion of the extracellular solution containing 100 µM barium chloride (BaCl_2_) added to the potassium current recorded before application of BaCl_2_.

### 5.4. Surface ECG Recordings

The surface lead II ECG signal was acquired using limb needle electrodes (MLA1213, AD Instruments, Sydney, Australia). The mice were placed in supine position on a homeothermic monitoring system (Harvard Apparatus, Holliston, MA, USA) to maintain the body temperature at 36–37 °C. Prior to the placement of limb electrodes, the mice were anesthetized using 200 mg/kg ketamine and 20 mg/kg xylazine i.p. along with 10 units of heparin. The needle electrodes were positioned under the skin, with the negative electrode on the right front paw, the positive electrode on left front paw, and the reference electrode on the left rear paw. ECG signals were recorded using an animal bioamplifier supplemented with PowerLab 8/35 and analyzed using LabChart Pro V.8 software (AD instruments). The signals were amplified and sampled at a rate of 1 kHz. An average of 1 min of the ECG signal was used in LabChart software to the calculate HR, P duration, PR interval, QRS duration, and QT interval. The QT interval was corrected for the RR interval according to the Mitchell formula: QTc = QT/√(RR × 10) [43].

### 5.5. Pseudo- and Intracardiac ECG Recordings on Langendorff-Perfused Hearts

Mice were anesthetized with 200 mg/kg ketamine and 20 mg/kg xylazine i.p. and sacrificed by cervical dislocation, after which the heart was rapidly excised. Hearts were retrogradely perfused on a Langendorff system using a modified Krebs–Henseleit buffer (KHB) containing (mmol/L): 116.5 NaCl_2_, 25 NaHCO_3_, 4.7 KCl, 1.2 KH_2_PO_4_, 1.2 MgSO_4_, 11.1 glucose, 1.5 CaCl_2_, 2 Na-pyruvate, and bubbled with 95% O_2_ and 5% CO_2_ at 37 °C. The pH levels of the buffer throughout the experiment with continuous bubbling ranged between 7.56 to 7.8. The perfusion pressure was maintained at 70 mmHg. Pseudo-ECGs were recorded using a pair of thin silver electrodes, with the negative end at the root of the aorta and the positive end on the apex of the ventricles. The data were acquired using Powerlab bioamplifiers and were low-pass-filtered at 5 kHz and high-pass-filtered at 10 kHz, then sampled at 1 k/s. For the initial 10 min, the heart was perfused with KHB buffer followed by 40 µg/mL mexiletine or vehicle control (methanol) dissolved in KHB. Pseudo-ECGs were continuously monitored throughout the experiment and later analyzed using LabChart Pro V.8 (AD Instruments, Australia). The shapes of the P wave, QRS complex, and QT region were defined as previously reported [44]. To quantify the ECG perturbation by mexiletine, we defined the QRS durations 2 min before the start of the mexiletine perfusion and 20 min after the start of mexiletine perfusion. We also studied atrioventricular (AV) and intraventricular (IV) conduction delays using a 1.1F (EPR-800, Millar Instruments, Houston, TX) octapolar intracardiac catheter inserted through the right atrium (RA) and advanced into the right ventricle (RV) on the explanted Langendorff-perfused heart. The proper catheter position was verified by visualization of at least six intracardiac electrocardiograms at the level of the right atria and ventricle overlaid by the P wave and QRS complex from the pseudo-ECGs on the same explanted heart. A representative ECG trace from a typical intracardiac recording configuration is shown in Figure 5A. To calculate the conduction delay, we measured the time delay between the first derivative negative peaks from different electrodes (CH 1-6) of the catheter. The distance between each electrode on the catheter was 1 mm. The AV delay was measured between CH 6 and 5 (1 mm distance), while the IV delay was measured between CH 4 and 1 (3 mm distance).

### 5.6. RNA Preparation and Quantitative RT-PCR

Total RNA samples were extracted from isolated ventricular and atrial tissue. RNA isolation was performed as reported previously [45] using TRIzol Reagent (Applied Biosystems, Foster City, CA, USA). PCR amplification was performed with TaqMan gene expression assay probes for mouse TRPM4 (Mm01205532-m1), Na_v_1.5 (Mm01342518-m1), and GAPDH (Mm99999915-g1). The quantitative expression analysis was performed with ViiA 7 Real-Time PCR System (Applied Biosystems, Foster City, CA, USA). Relative quantification was performed using the comparative threshold (Ct) method (ΔCt) after determining the Ct values for the reference (GAPDH) and the target genes (TRPM4 or Na_v_1.5) for each sample set.

### 5.7. Western Blot

Proteins from freshly isolated myocytes were prepared by lysing in 0.3 mL ice-cold lysis buffer (50 mM HEPES, 150 mM NaCl, 1 mM EGTA, 10% glycerol, 1.5 mM MgCl_2_, 10 mM NEM, cOmplete protease inhibitor (Roche diagnostics, GmbH ref.11697498001) on a rotating wheel at 4 °C overnight. The obtained homogenate was centrifuged at 3000× *g* at 4 °C for 15 min to remove large cellular fragments. Next, 50 µL of the supernatant was used for Na_v_1.5 staining with total lysate. The remaining supernatant was ultra-centrifuged at 200,000× *g* for 45 min in saccharose buffer (1 mol/L saccharose, 1 mol/L HEPES, and cOmplete protease inhibitor). The pellets containing total membrane fractions were solubilized in a cold saccharose buffer. Protein concentrations were determined by Bradford assay using bovine serum albumin (BSA) as a standard. About 80 µg of protein from ventricular myocytes and 25 µg from atrial myocytes were dissolved with 4X LDS sample buffer and 100 mM DTT. The sample was denatured at 37 °C for 30 min and separated in 6% or 9% SDS-PAGE gel to detect Na_v_1.5 or TRPM4, respectively. Proteins were transferred from the gel to a nitrocellulose membrane (Bio-Rad, Hercules, CA, USA). The respective proteins were detected with custom rabbit polyclonal antibodies (Na_v_1.5 epitope: DRLPKSDSEDGPRALNQLS (Pineda Antibody Service, Berlin, Germany); TRPM4 epitope: VGPEKEQSWIPKIFRKKVC (Pineda Antibody Service, Berlin, Germany)). Calnexin (Sigma-Aldrich, St. Louis, MO, USA., Cat No: C4731) was used to normalize TRPM4 and Na_v_1.5 protein band intensities. All of the Western blots were quantified using Image Studio Lite software (LI-COR Biosciences, Lincoln, NE, USA).

### 5.8. Immunoprecipitation Studies

HEK-293 cells transfected with appropriate plasmids were lysed 48 h after transfection for 1 h at 4 °C. The protein was quantified and 50 µg was used as the input fraction. Then, 2 mg of the total protein was further incubated with anti-FLAG Dynabeads (Sigma M8823) for 1 h at 4 °C. Using a magnetic holder, unbound protein was removed, washed with lysis buffer, then the bound protein was eluted into sample buffer. The input and the immunoprecipitated fraction were further analyzed by Western blot. Proteins were visualized by anti-Na_v_1.5 or anti-TRPM4 (both Pineda Antibody Service, Berlin, Germany), anti-TRPM5 (Abcam, ab154788), or anti-Na/K pump (Abcam, ab7671).

### 5.9. Statistical Analyses

Data are presented as means ± SEMs unless otherwise specified. The statistical significance of differences between two means was determined using Student’s *t*-test if *n* ≥ 5. *P* values ≤ 0.05 were accepted as significant and represented as *#* in respective figure panels. The experimenter and person analyzing the data were blindfolded to the genotype of the mice. The number of mice and the number of cells used for each experiment are represented as *N* and *n,* respectively, in the figure legends.

## Figures and Tables

**Table 1:** Action potential parameters in atrial and ventricular myocytes from WT or *Trpm4*^-/-^ animals. Data are represented as means ± SD. Note: #: *p* ≤ 0.05, WT vs. *Trpm4^−/−^*.

	Atrial		Ventricular	
	WT	*Trpm4* ^−/−^	WT	*Trpm4* ^−/−^
RMP	−71.0 ± 3.6	−68.9 ± 2.5 #	−81.1 ± 4.6	−82.9 ± 5.1
AMP	107.0 ± 14.2	106.5 ± 12.3	112.0 ± 7.8	101.0 ± 11.5
V_max_ (mV/ms)	107.7 ± 64.0	102.2 ± 48.0	115.8 ± 30.0	89.7 ± 28.3 #
APD 30	8.8 ± 4.3	9.0 ± 5.7	4.3 ± 3.2	4.7 ± 2.2
APD 50	18.0 ± 8.7	18.5 ± 11.8	8.5 ± 6.0	8.6 ± 3.9
APD 90	71.5 ± 19.7	77.8 ± 26.9	58.1 ± 30.3	52.7 ± 12.4

**Table 2:** Summary of *I*_Na_ patch clamp data. (#: *p* ≤ 0.05, WT vs. *Trpm4^−/−^*).

	Atrial		Ventricular	
	WT	*Trpm4* ^−/−^	WT	*Trpm4* ^−/−^
Cm (pF)	62.2 ± 3.8	50.8 ± 2.4 #	187.7 ± 16.7	164.5 ± 10.9
*I_Na_*-peak density (pA/pF)	−48.0 ± 2.3	−36.3 ± 2.6 #	−36.0 ± 4.4	−25.6 ± 1.6 #
V_1/2_ of activation (mV)	−38.6 ± 1.4	−34.3 ± 0.9 #	−34.7 ± 0.1	−32.4 ± 0.1
*k* of activation	4.0 ± 0.2	5.2 ± 0.2 #	5.6 ± 0.1	5.5 ± 0.1
V_1/2_ of inactivation (mV)	−75.0 ± 1.9	−76.2 ± 1.4	−75.5 ± 0.1	−74.9 ± 0.9
k of inactivation	6.2 ± 0.1	6.1 ± 0.1	6.0 ± 0.1	6.6 ± 0.1

**Table 3:** Summary of surface and pseudo-ECG parameters from anesthetized mice. Note: #: *p* ≤ 0.05, WT vs. *Trpm4^−/−^.*

	Surface ECG		Pseudo-ECG	
	WT	*Trpm4* ^−/−^	WT	*Trpm4* ^−/−^
P dur. (ms)	12.1 ± 2.1	14.1 ±2.0	13.8 ± 0.7	19.0 ± 0.9 #
PR (ms)	47.1 ± 0.9	43.3 ± 2.6	45.4 ± 2.2	48.8 ± 3.0
QRS (ms)	10.2 ± 0.2	10.4 ± 0.5	9.1 ± 0.4	10.3 ± 0.5
QT (ms)	56.3 ± 2.1	65.1 ± 4.1	80.5 ± 12.4	99.1 ± 10.3
QTc (ms)	43.7 ± 2.9	44.2 ± 3.3	52.0 ± 7.3	61.8 ± 6.6
HR (bpm)	361 ± 20.0	280 ± 19.0 #	246 ± 11.9	230 ± 8.7

## Data Availability

All data are contained within this manuscript.

## Supplementary Materials

The following are available online at https://www.mdpi.com/article/10.3390/ijms22073401/s1.
