# Supplementary material for: Deletion of Trpm4 Alters the Function of the Nav1.5 Channel in Murine Cardiac Myocytes
Source: Int J Mol Sci. 2021 Mar 26;22(7):3401. doi: 10.3390/ijms22073401 (PMC8037196; doi:10.3390/ijms22073401)
Supplement: Supplementary file 1 [file ijms-22-03401-s001.pdf]

# Supplementary Figures

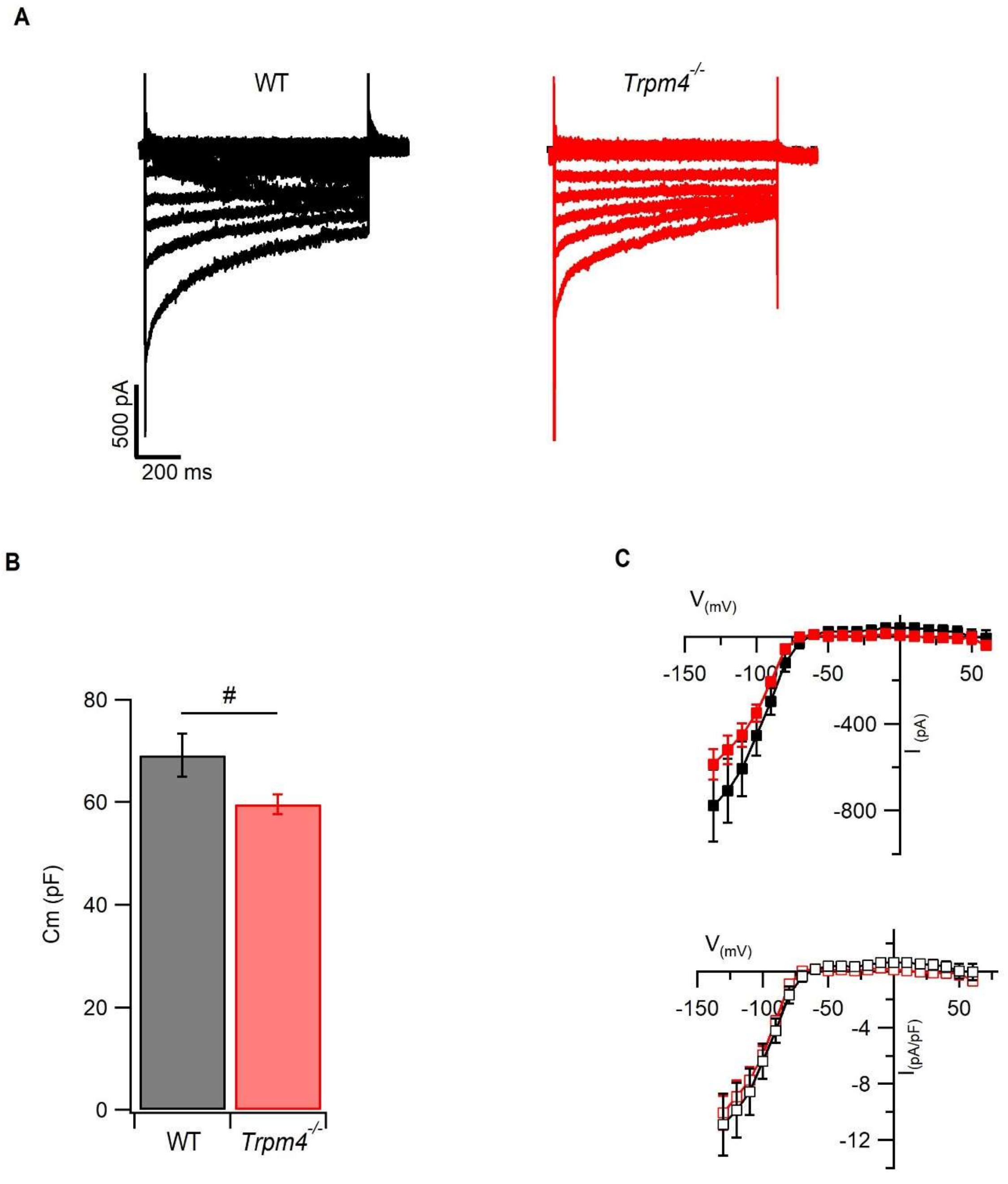


**<u>Supplementary Figure S1</u>:** *$I_{K1}$ current measurement in atrial myocytes.* **A)** Representative voltage-clamp current traces for assessment of $Ba^{+}$ sensitive inward-rectifier $K^{+}$ current $I_{K1}$ in WT or *Trpm4*$^{-/-}$ atrial cardiomyocytes**. B)** Average cell capacitance measured and compared between both genotypes. **C)** Current-voltage relationship of peak $I_{K1}$ represented either as current or current density normalized to cell capacitance compared between WT ($N = 3$, $n = 18$) and *Trpm4*$^{-/-}$ ($N = 3$, $n = 19$) mice.

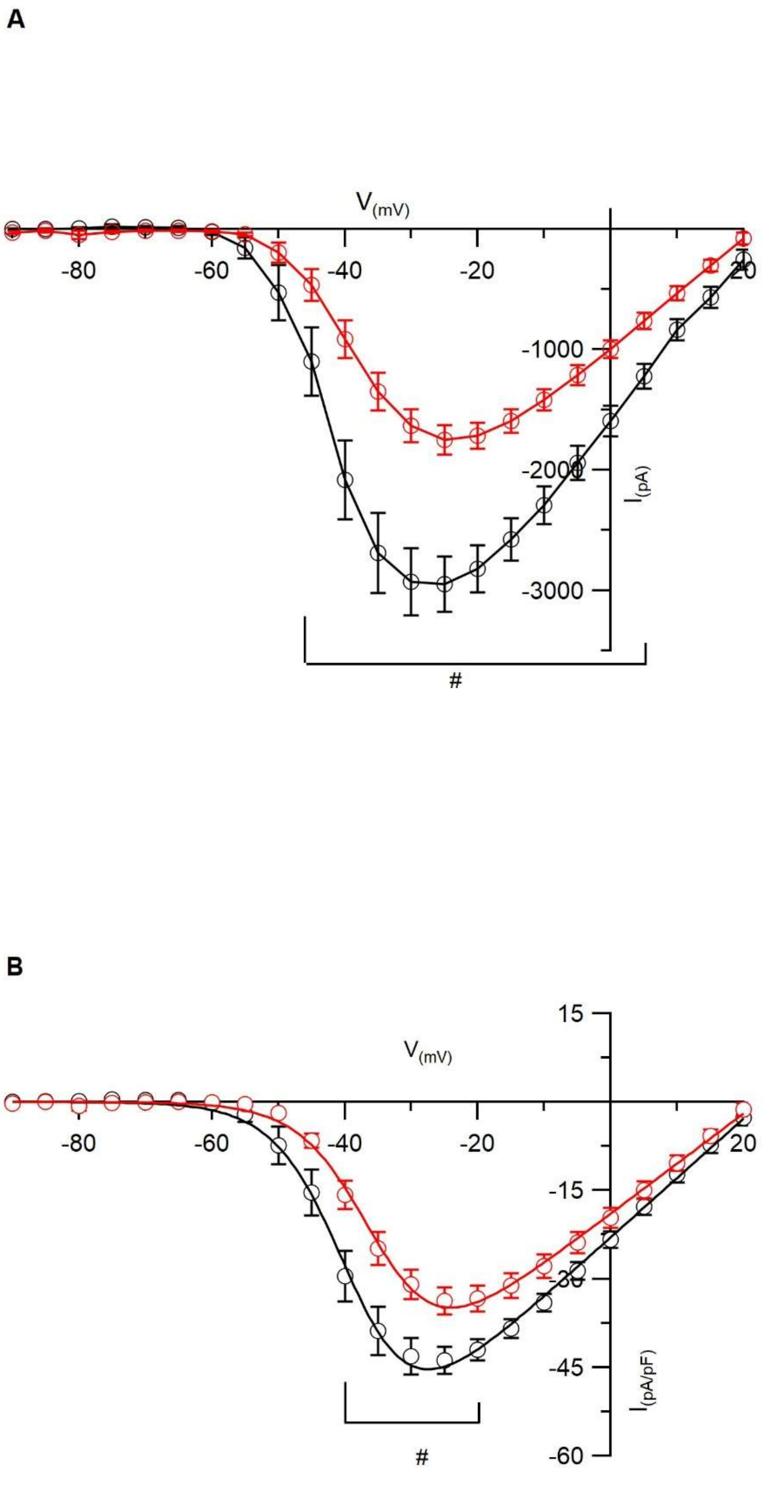


**Supplementary Figure S2:** $Na_V1.5$ current (A) and current density normalized to cell capacitance (B) of WT (black) and *Trpm4*$^{-/-}$ (red) mice ($N = 5$; $n = 20$ for WT, $n = 22$ for *Trpm4*$^{-/-}$ ; #: $p < 0.05$, WT vs *Trpm4*$^{-/-}$ at a given voltage)

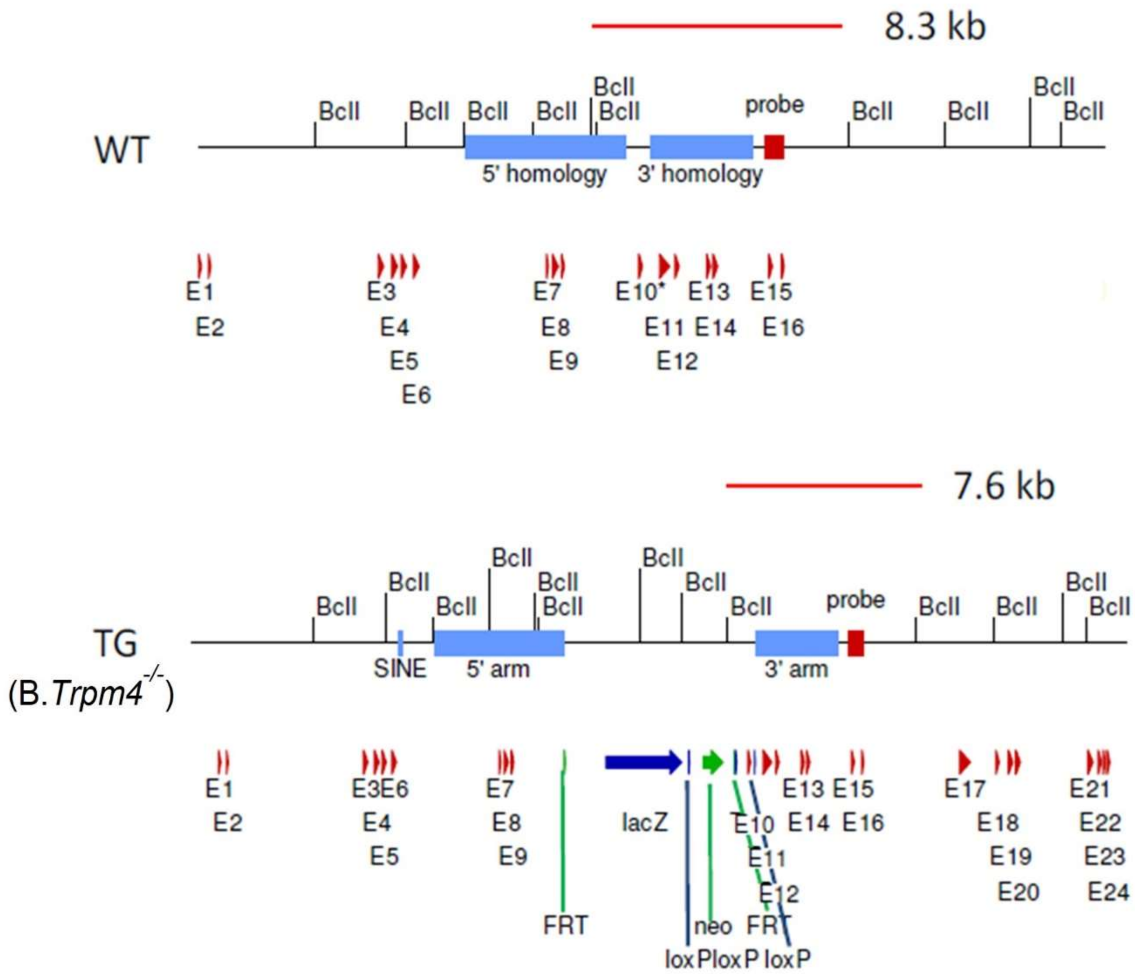


**Supplementary Figure S3:** *Generation of Trpm4 knock-out mouse strain.* Schematic drawing of the targeted *Trpm4* locus. Two alleles of the *Trpm4* locus are depicted: the wild type locus (WT) and the targeted allele (TG). After homologous recombination, three loxP sites (blue arrows) are inserted into the genome flanking exon 10 of Trpm4 (red arrows). Additionally, an FRT flanked neomycin (green) / lacZ (blue) cassette is inserted upstream of exon 10. NB: this B.*Trpm4*$^{-/-}$ strain (Generated by PolyGene AG, Rümlang, Switzerland) was backcrossed on a C57BL6/J background. In all experiments, male *Trpm4*$^{-/-}$ mice and wildtype (WT) littermates aged 12-15 weeks were used.

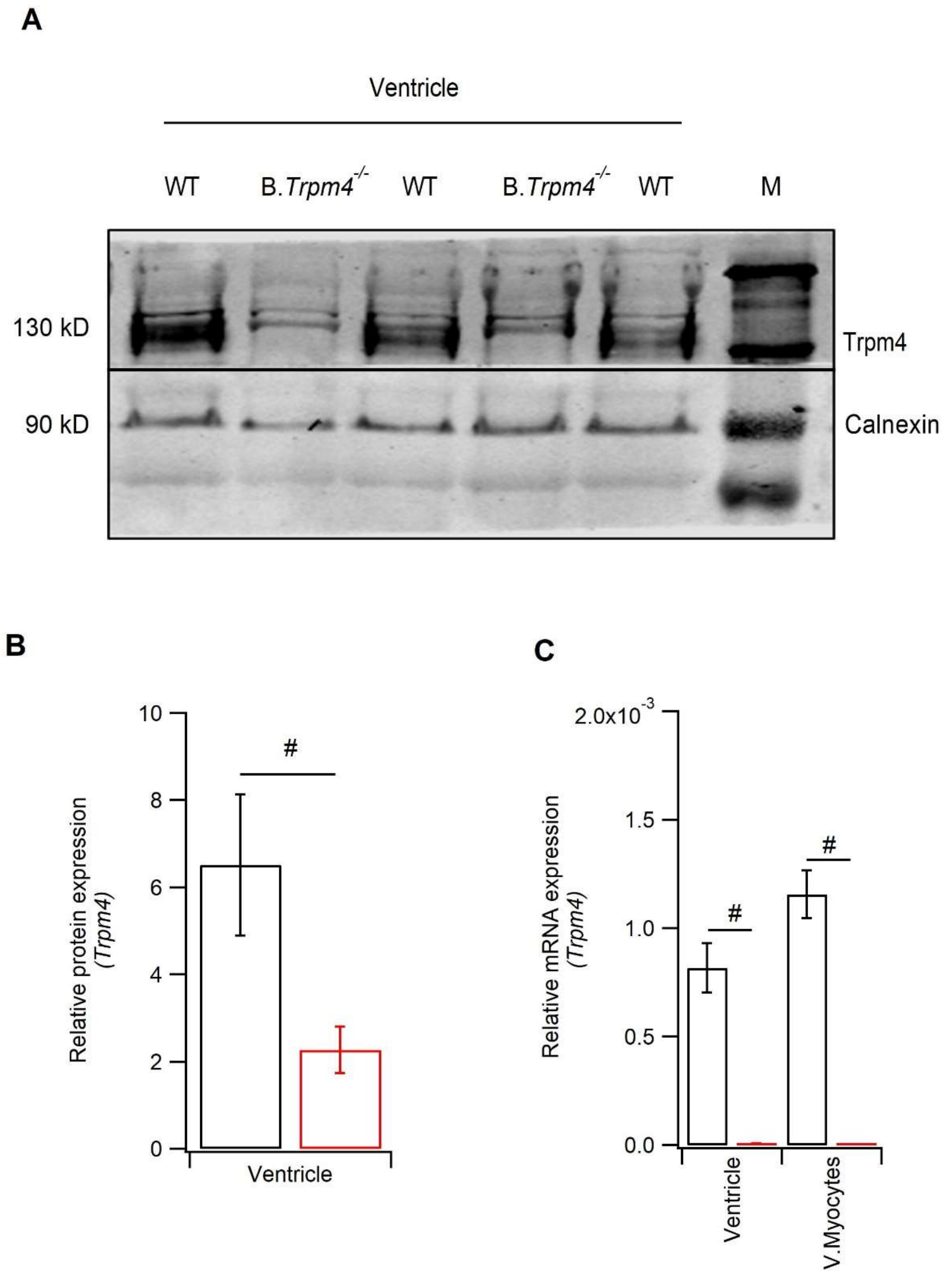


<u>**Supplementary Figure S4:**</u> *Expression of Trpm4 in mouse ventricle.* (**A**) Representative immunoblots of TRPM4 total protein expression in mouse ventricular tissue. Quantification of relative protein (N =7) **(B)** and mRNA (N =4) **(C)** in either ventricular tissue or isolated ventricular myocytes. (#: $p < 0.05$ for WT (black) vs. B. *Trpm4*$^{-/-}$ (red))

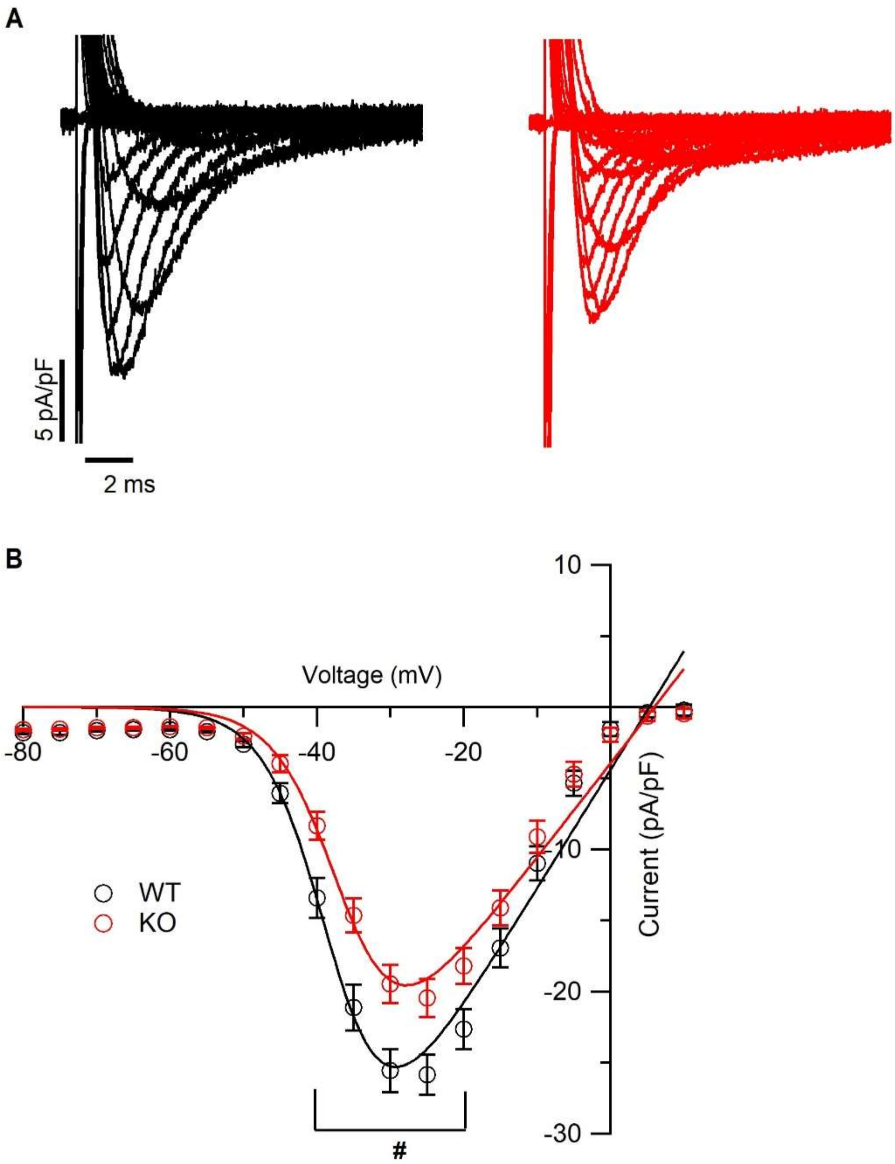


**<u>Supplementary Figure S5</u>:** *Na⁺ current recordings from isolated ventricular myocytes.* **A)** Representative voltage-clamp current traces for peak $Na^+$ current measurements in WT (black) and B.*Trpm4*$^{-/-}$ (red) isolated ventricular myocytes. **B)** Current-voltage relationship of peak $Na^+$ current represented as current density in WT (*N* = 6, *n* = 43) and B.*Trpm4*$^{-/-}$ (N = 6, n = 38) mice. (#: $p < 0.05$ at given voltages for WT vs. B.*Trpm4*$^{-/-}$)

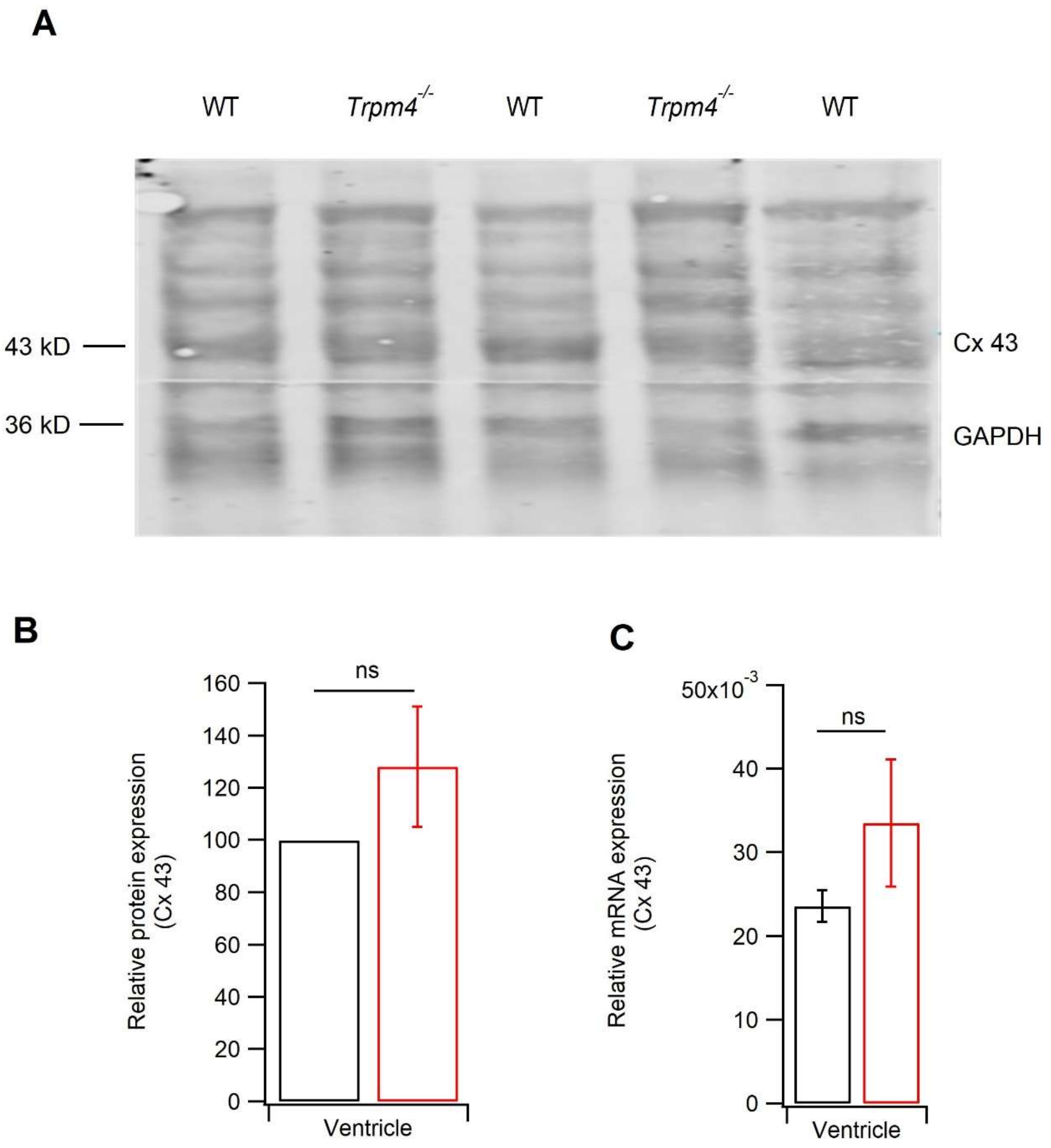


**<u>Supplementary Figure S6</u>:** *Connexin 43 (Cx 43) expression in WT or Trpm4$^{-/-}$*. (**A**) Representative immunoblots of Cx43 total protein expression in mouse ventricular tissue; WT (black), *Trpm4$^{-/-}$* (red). Quantification of relative protein **(B)** and mRNA **(C)** in ventricular tissue. (N = 4; ns – not significant)

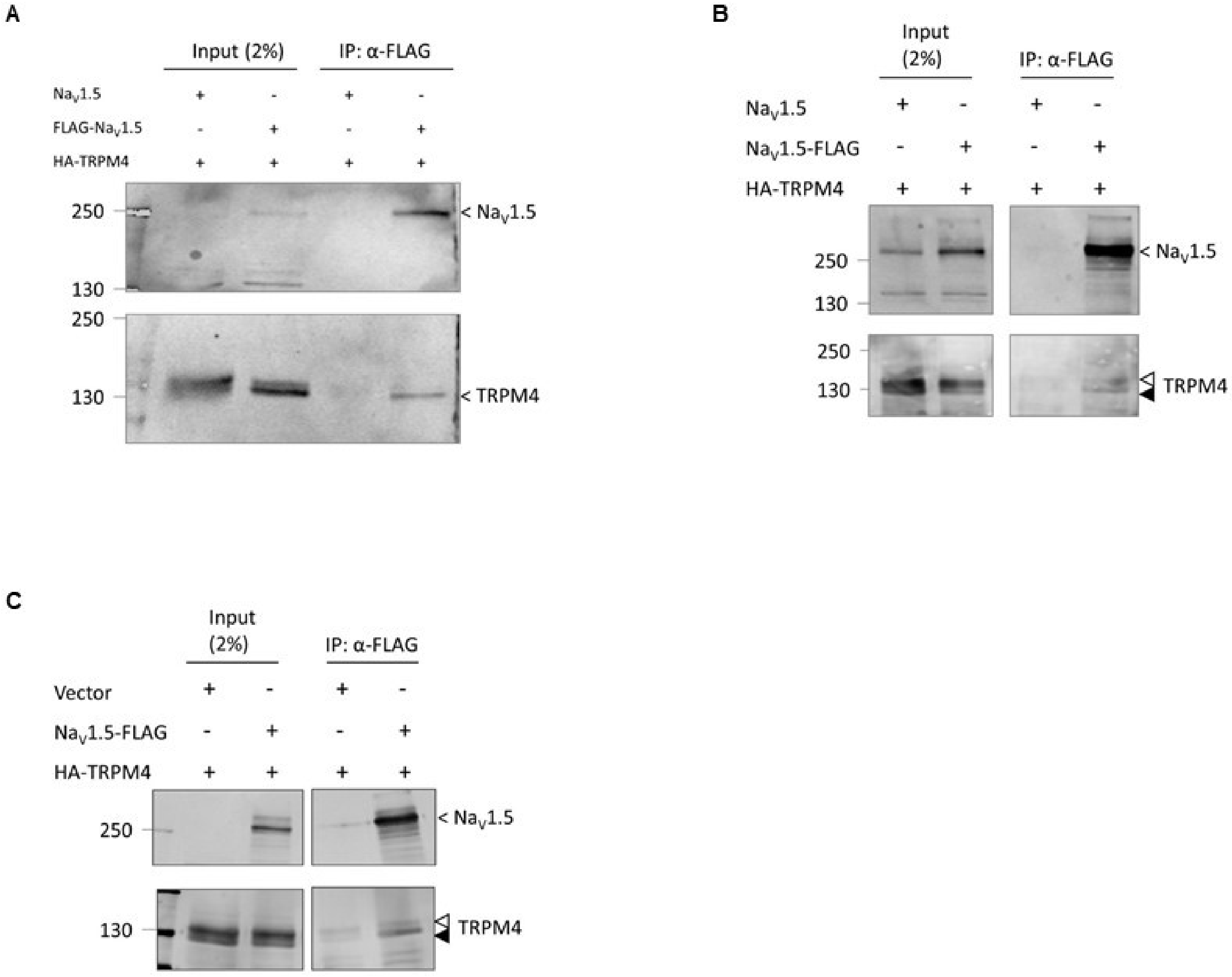


**<u>Supplementary Figure S7</u>:** *A fraction of TRPM4 binds to Na$_v$1.5 :* **A, B** and **C** are immunoblots showing biological replicates of co-immunoprecipitation between TRPM4 and Na$_v$1.5 in HEK-293 cells. Input signals confirm the expression of Na$_v$1.5 with or without FLAG tag and TRPM4. In the immunoprecipitated (IP) fraction, the signal is observed only in the presence of FLAG-Na$_v$1.5 and TRPM4.
